# Can Metabolic Disorders in Aging Men Contribute to Prostatic Hyperplasia Eligible for Transurethral Resection of the Prostate (TURP)?

**DOI:** 10.3390/ijerph120303327

**Published:** 2015-03-19

**Authors:** Aleksandra Rył, Iwona Rotter, Marcin Słojewski, Adriana Jędrzychowska, Zuzanna Marcinowska, Marta Grabowska, Maria Laszczyńska

**Affiliations:** 1 Department of Histology and Developmental Biology, Pomeranian Medical University, Szczecin 71-210, Poland; E-Mails: ryl.ola@poczta.gmail.com (A.R.); grabowska.marta.anna@gmail.com (M.G.); maria@laszczynska.pl (M.L.); 2 Laboratory of Rehabilitation Medicine, Pomeranian Medical University, Szczecin 71-210, Poland; 3 Department of Urology and Urological Oncology, Pomeranian Medical University, Szczecin 71-111, Poland; E-Mail: mslojewski@gmail.com; 4 Laboratory Diagnostics and Molecular Medicine, Department of Laboratory Medicine, Pomeranian Medical University, Szczecin 71-111, Poland; E-Mails: adriana.jedrzychowska@gmail.com (A.J.); zuzia26@o2.pl (Z.M.)

**Keywords:** benign prostatic hyperplasia, metabolic syndrome

## Abstract

*Purpose*: The aim of this study was to evaluate the incidence and severity of metabolic disorders occurring in the metabolic syndrome in patients with benign prostatic hyperplasia eligible for surgical treatment. *Methods*: The study group consisted men with diagnosed benign prostatic hyperplasia. The control group consisted patients recruited from basic health care units. Abdominal circumference, body weight and blood serum metabolic parameters were determined in the experimental and control groups. The concentrations of glucose were determined, as well as total cholesterol (ChT), low-density lipoprotein (LDL), high density lipoprotein (HDL) and triglycerides (TAG), by spectrophotometric method using reagent kits. *Results*: In the study group 91 (60.3%) cases of metabolic syndrome (MetS) were diagnosed, while in the control group 71 (46.1%) men met the diagnostic criteria for this syndrome (*p* = 0.018). The analysis shows a relationship between MetS in patients with BPH and concentration glucose, ChT, LDL, HDL, systolic blood pressure and diastolic blood pressure. We found no significant statistical relationship between body weight, abdominal circumference and concentration TAG, hypertension in patients and controls. *Conclusions*: in the study presented in this article, statistically significant relationships between BPH and the diagnostic parameters of the metabolic syndrome were demonstrated. These results indicate to the necessity of the modification of the lifestyle, taking preventive measures in diabetes, and evaluation of lipid metabolism disorders. It is recommended to assess symptoms that may suggest BPH (as a manifestation of LUTS) in men over 50 years of age with diagnoses of metabolic disorders (including MetS), and provide them with specialist urological care in order to prevent surgical treatment of the prostate.

## 1. Introduction

Metabolic syndrome (MetS) is a clinical term proposed by the American endocrinologist Gerald Reaven in 1988 [1]. It is also known as metabolic syndrome X, dysmetabolic syndrome, polymetabolic syndrome, insulin resistance syndrome, Reaven syndrome and deadly quartet syndrome*.* Due to its grave socio-economic implications and the growing incidence, it is increasingly often mentioned as a global epidemic [1,2,3].

MetS can be established based on parallel criteria from the International Diabetes Federation (IDF) and the National Cholesterol Education Program—Adult Treatment Panel III (NCEP-ATP III) from 2001, with subsequent modification in 2004 [4,5,6]. In general, they define metabolic syndrome as the concurrence of atherogenic dyslipidemia, disrupted glucose metabolism, hypertension and obesity, all contributing to an increased risk of cardiovascular disease; mainly ischemic heart disease and type 2 diabetes [7,8,9]. In addition, patients diagnosed with metabolic syndrome also often suffer from thrombotic conditions and inflammation [10].

The concept of metabolic syndrome as a distinct disease has both its supporters [4,11] and opponents [10,12]. The latter include the American Diabetes Association and the European Association for the Study of Diabetes who question the rationale for distinguishing metabolic syndrome as a distinct disease. They point to the lack of difference between the treatment of MetS and disorders used as its diagnostic criteria, the unclear pathogenesis and the lack of uniform diagnostic definition [13].

Epidemiological data indicate that about 25% of middle-aged people in developed countries show the symptoms of metabolic syndrome [14,15]. The prevalence of metabolic syndrome in Poland is shown by population-based surveys, such as Hypertension in Poland Plus, Plus Lipid Disorders and Diabetes (NATPOL PLUS) [16] and the Multicenter Study of National Population Health (WOBASZ 1) [17]. The NATPOL PLUS surveyed 2329 people over 18 years of age who had been diagnosed MetS on the basis of NCEP-ATP III criteria.

The surveys show the presence of metabolic syndrome in 20% of the Polish population [17], with its incidence increasing with age. The symptoms of MetS (according to WOBASZ 1) were observed in 9.7% of men aged 20–39 years, 28.4% of men aged 40–59 years, and in 34.5% of those aged aged 60–74 [16]. The incidence of its diagnostic criteria in Polish men are as follows: hypertension in 69.2% of men, abdominal obesity 28.0%, fasting hyperglycemia 19.6%, decreased HDL 9.8% and triglycerides above the norm in 33.8% [17]. This is in line with data from the Central Statistical Office in Poland showing a high male mortality in Poland. Men account for 53% of all recorded deaths in Poland, with nearly half of them caused by cardiovascular diseases [18].

Abdominal obesity is one of the key factors in the diagnosis of MetS (according to IDF criteria). Research indicates that early abdominal obesity precedes the other diagnostic factors of MetS [19]; the pro-inflammatory action of the visceral fat (production of adipokines) is a predisposing factor for the occurrence of the other factors [20]. For example, the relationship between obesity and insulin resistance is widely described in literature [21,22,23]. The clinical consequences of insulin resistance include type 2 diabetes or pathological changes in the cardiovascular system. Patients with impaired insulin sensitivity are also likely to develop impaired glucose tolerance and impaired fasting glucose [24]. The mathematical description of insulin sensitivity is provided by the Homeostatic Model Assessment (HOMA), developed by Matthew *et al.* in 1985 [25].

Hypertension is the most common criterion taken into account in the diagnosis of MetS. According to various studies, about 60% of Poles aged 50+ have trouble maintaining normal blood pressure [26,27]. One of the other MetS factors, impaired lipid metabolism, is one of the most significant risk factors for cardiovascular disease [28]. According to some estimates, primary hypercholesterolemia may affect 10% of the Polish population, and atherogenic dyslipidemia as much as 30% [29].

Epidemiological and histopathologic research provides clinical evidence of a correlation between the concurrence of metabolic disorders within the metabolic syndrome and the pathogenesis of benign prostatic hyperplasia (BPH) [30,31]. The induction of inflammatory states in the prostatic cells seems to be an important linking factor between BPH and metabolic disorders (espeially those associated with the disturbed carbohydrate and lipid metabolism) [32,33,34]. Studies conducted on animal models show that the purposeful induction of factors enabling the diagnosis of MetS in animals, entails the development of histopathological lesions in the prostate gland [35]. Benign prostatic hyperplasia is a most common disease in men and incidence increases with the age of patients. Hyperplasia is observed in about 20% of men in the fourth decade of life, and up to approximately 80% after 80 years of age [36,37].

The aim of this study was to evaluate the incidence and severity of metabolic disorders occurring in the metabolic syndrome in patients with benign prostatic hyperplasia eligible for surgical treatment.

## 2. Materials and Methods

### 2.1. The Study Group

The study was conducted in 305 men aged 50 – 75. The study group consisted of 151 (mean age 67.3 ± 8.3 ± SD) men with diagnosed and surgically treated benign prostatic hyperplasia. These were patients accepted for transurethral resection of the prostate (TURP) at the Department of Urology and Urological Oncology at the Pomeranian Medical University in Szczecin. The patients in the study group took the finasteride (5 mg orally once a day).

The control group consisted of 154 patients (60.3 ± 6.2) recruited from basic health care units in Szczecin. Men in the control group called patients according to the WHO definition: term “patient” can be used when referring to any person regardless of whether he/she is healthy or ill and using health care services [38].

The patients in the control group did not report any problems with the lower urinary tract, did not take the antagonists of 5α-reductase inhibitor receptor or α1-adrenergic receptor and were not under the care of a urologist.

The study groups and control were divided into four groups:

1. with benign prostatic hyperplasia and with no evidence of metabolic syndrome
2. with benign prostatic hyperplasia and diagnosed with metabolic syndrome
3. with no benign prostatic hyperplasia and with no evidence of metabolic syndrome
4. with no benign prostate hyperplasia and diagnosed with metabolic syndrome

Metabolic syndrome was diagnosed according to the IDF criteria of 2005 (abdominal circumference ≥ 94 cm, and at least two of the following deviations: glycemia on an empty stomach ≥ 100 mg/dL or treatment for type 2 diabetes, arterial blood pressure ≥ 130/85 mm·Hg or treatment for hypertension, the level of HDL cholesterol < 40 mg/dL in men or treatment for dyslipidemia, the levels of triglycerides (TAG) ≥ 150 mg/dL or treatment for dyslipidemia) [4].

### 2.2. Clinical Trials

Abdominal circumference, body weight and blood serum metabolic parameters were determined in the experimental and control groups. In addition, the patients filled in a questionnaire with sociodemographic data, a late-onset hypogonadism questionnaire by Morley’ and a standardized International Prostate Symptom Score (IPSS). Patients with active cancer, those treated with steroid drugs and neuroleptics, with active alcoholism, and with liver and thyroid disease, were excluded from the study.

### 2.3. Diagnostic Investigations

Nine milliliters of venous blood samples were taken from fasting patients’ cubital veins. After centrifugation, serum was transferred to Eppendorf tubes and stored in a freezer at −70 ^°^C for a no longer than 3 months. The concentrations of glucose were determined, as well as ChT, LDL, HDL and TAG, by spectrophotometric method using reagent kits (Biolabo, Aqua-Med, Lodz). Insulin levels were determined by ELISA using reagent kits (DRG Medtec, Warsaw). HOMA coefficient was calculated based on the formula: HOMA = fasting glucose level (mmol/L) × fasting insulin level (µU/mL) / 22.5. Under physiological conditions, the indicator value should be 1 or less. A higher HOMA value indicates the presence of insulin resistance.

## 3. Statistical Analysis

Data analysis was performed using SPSS Statistics version 14.0 software for Windows (SPSS Inc., Chicago, IL, USA). Basic statistics included determination of the group sizes, minima, maxima, first and third quartiles, medians, quartile ranges, arithmetic means and standard deviations. The normality of distribution was tested using a Shapiro-Wilk test. Quantitative data was analyzed using non-parametric Mann-Whitney U tests, and data measured on a dichotomous scale were analyzed using a chi-square test of independence with Yates’ correction. The results were deemed significant at *p* < 0.05.

### Permission to Conduct Research

The study was approved by the Bioethics Committee at the Pomeranian University in Szczecin, approval number KB-0012/132/12.

## 4. Results

Patients eligible for transurethral resection of the prostate were significantly more likely to have MetS (*p* = 0.018), diabetes (*p* = 0.0477) compared to the controls with no prostate hyperplasia. We found no statistical relationship between the occurrence of benign prostatic hyperplasia and the incidence of hypertension (Table 1).

In the patients with benign prostatic hyperplasia (Table 2) 91 (60.3%) cases of MetS were diagnosed, 57.0% of patients suffered from hypertension, 23.8% of patients reported a history of diagnosed and pharmacologically treated diabetes. In the patients with benign prostatic hyperplasia of average abdominal circumference was 99.2 ± 9.4 cm (X̅ ± SD). The mean body weight of the studied patients was 83.0 ± 13.2 kg. In patients with BPH were the following concentrations: average blood plasma glucose was 110.4 ± 38 mg/dL, HOMA was 5.4, mean systolic 119.9 ± 13.8 and diastolic 77.4 ± 9.8 blood, total cholesterol levels 190.5 ± 59.0 mg/dL, HDL 35.1 ± 10.3 mg/dL, LDL 126.7 ± 56.4 mg/dL.

In the patients without benign prostatic hyperplasia (Table 3) 71 (46.1%) cases of MetS were diagnosed, 52.6% of patients suffered from hypertension, 14.3% of patients reported a history of diagnosed and pharmacologically treated diabetes. In the patients without benign prostatic hyperplasia of average abdominal circumference was 100.6 ± 10, while body weight was 84.4 ± 3.6 kg. In patients without BPH were the following concentrations: average blood plasma glucose was 118.1 ± 35.7 mg/dL, HOMA was 3.74, mean systolic 135.2 ± 21.1 and diastolic 84.6 ± 11.3 blood, total cholesterol levels was 216.3 ± 55.5 mg/dL, HDL 50.9 ± 13.7 mg/dL, LDL 136.8 ± 50.4 mg/dL, TAG 146.2 ± 13.0 mg/dL.

We found significant statistical relationship (Table 4) between average blood plasma glucose (*p* = 0.0001), mean systolic (*p* ≤ 0.0001) and diastolic (*p* ≤ 0.0001) blood, total cholesterol levels (*p* = 0.00014), HDL *p* ≤ 0.0001, LDL *p* = 0.021, TAG 144.9 ± 90.8 mg/dL in patients in study and controls group. We found no significant statistical relationship between body weight, abdominal circumference, HOMA, TAG levels (*p* = 0.1) in patients in study and controls group.

**Table 1:** Metabolic diseases in patients with and without benign prostatic hyperplasia.

Disease	Patients with Prostatic Hyperplasia (n = 151)		Patients without Prostate Hyperplasia (n = 154)		Statistical Significance (*p*)
	Number of Patients	%	Number of Patients	%	
Diabetes	36	23.8	22	14.3	0.0477 *
Hypertension	86	57	81	52.6	0.52
Metabolic syndrome	91	60.3	71	46.1	0.018 *

* *p* ≤ 0.05.

**Table 2:** Selected statistical parameters of the diagnostic criteria of metabolic syndrome and additional parameters in patients with benign prostatic hyperplasia.

Patients with Benign Prostatic Hyperplasia (n = 151)						
Diagnostic Criteria for Metabolic Syndrome						
Parameters	Abdominal Circumference (cm)	Glucose (mg/dL)	Systolic Blood Pressure (mm·Hg)	Diastolic Blood Pressure (mm·Hg)	HDL (mg/dL)	TAG (mg/dL)
min–max	79–125	43–375	90–160	55–90	20.7–75	57.4–609.1
Q1–Q3	92–105	94–114	110–120	70–80	28–38.2	99.1–160.8
me (Q)	98 (6.5)	101 (10)	120 (5)	80 (5)	33.2 (5.1)	134.4 (30.8)
X ± SD	99.2 ± 9.4	110.4 ± 38	119.9 ± 13.8	77.4 ± 9.8	35.1 ± 10.3	146.2 ± 13.0
**Other Criteria**						
**Parameters**	**Age (years)**	**Body Weight (kg)**	**BMI (kg/m^2^)**	**Total Cholesterol (mg/dL)**	**LDL (mg/dL)**	**HOMA (mg/dL)**
min-max	52–91	51.5–120	18.7–39.2	83.8–454.9	18.1–405.3	0.11–27.87
Q1-Q3	61–73	75–91	24.7–30.1	149.6–217.2	89.6–156.1	1.38–7.09
me (Q)	65 (6)	81 (8.0)	27 (2.7)	180.5 (33.8)	113.8 (66.5)	2.69 (2.9)
X ± SD	67.3 ± 8.3	83.0 ± 13.2	27.7 ± 4.1	190.5 ± 59	126.7 ± 56.4	5.4 ± 6.14

n—the size of the group; min-max—min, max; Q1–Q3—the first and third quartile; me (Q)—median (quartile deviation); X ± SD—arithmetic mean ± standard deviation.

**Table 3:** Selected statistical parameters of the diagnostic criteria of metabolic syndrome and additional parameters in patients without benign prostatic hyperplasia.

Patients without Benign Prostatic Hyperplasia (n = 154)						
Diagnostic Criteria for Metabolic Syndrome						
Parameters	Abdominal Circumference (cm)	Glucose (mg/dL)	Systolic Blood Pressure (mm Hg)	Diastolic Blood Pressure (mm·Hg)	HDL (mg/dL)	TAG (mg/dL)
min–max	75–140	52.8–346.6	80–220	50–120	19.3–94.8	37.3–58.4
Q1–Q3	94–106	98.8–127.8	120–150	80–90	40.7–60.2	84.2–118.2
me (Q)	99 (6)	109 (14.5)	135 (5)	80 (5)	49.7 (9.8)	121.2 (51.98)
X± SD	100.6 ± 10	118.1 ± 35.7	135.2 ± 21.1	84.6 ± 11.3	50.9 ± 13.7	144.9 ± 90.8
**Other Criteria**						
**Parameters**	**Age (years)**	**Body Weight (kg)**	**BMI (kg/m^2^)**	**Total cholesterol (mg/dL)**	**LDL (mg/dL)**	**HOMA (mg/dL)**
min–max	50–74	54–135	20–43.6	96.1–351.6	29.3–287.2	0.81–9.17
Q1–Q3	55–65	76–90	25.2–29.4	176.5–249.1	102.8–167.9	2.29–4.84
me (Q)	60 (5)	63 (7)	27 (2.1)	210.2 (36.3)	131.7 (33)	3.13 (1.27)
X± SD	60.3 ± 6.2	84.4 ± 3.6	27.4 ± 3.6	216.3 ± 55.5	136.8 ± 50.4	3.74 ± 1.97

n—the size of the group; min-max—min, max; Q1–Q3—the first and third quartile; me (Q)—median (quartile deviation); X ± SD—arithmetic mean ± standard deviation.

**Table 4:** The statistical significance between the parameters in patients with and without benign prostatic hyperplasia.

Parameters		Statistical Significance (*p*)
Diagnostic criteria for metabolic syndrome	Abdominal circumference (cm)	0.18
	Glucose (mg/dL)	0.0001 *
	Systolic blood pressure (mm·Hg)	≤0.0001 *
	Diastolic blood pressure (mm·Hg)	≤0.0001 *
	HDL (mg/dL)	≤0.0001 *
	TAG (mg/dL)	0.1
Other criteria	Age (years)	0.0001 *
	Body weight (kg)	0.26
	BMI (kg/m^2^)	0.86
	Total cholesterol (mg/dL)	0.00014 *
	LDL (mg/dL)	0.021 *
	HOMA (mg/dL)	0.300

* *p* ≤ 0.05.

## 5. Discussion

A new approach to the epidemiology of benign prostatic hyperplasia consists in searching for the common etiology of BPH and cardiovascular diseases [39]. Metabolic disorders and patient lifestyle may not only result in cardiovascular diseases but may also adversely affect the urinary tract.

Both MetS and BPH are characteristic in the elderly and the prevalence increases with age. The results of our study indicate that men with benign prostatic hyperplasia eligible for surgical treatment were more likely to have MetS than men without BPH. A similar relationship was noticed by Hammarsten *et al.* in 1998 [40]. Those authors studied men living in Sweden who were diagnosed with BPH, and found that people with metabolic syndrome had increased prostate volume (49 mL) than patients with BPH and with no evidence of metabolic syndrome (28.5 mL).

Benign prostatic hyperplasia is a serious public health problem and research on the quality of life of patients is significant for correct diagnosis. Currently, symptoms caused by prostatic hyperplasia are estimated based on scores in the IPSS and Quality of Life (QoL) questionnaires. Prostate volume is an important parameter for patient quality of life and ailments caused by prostatic hyperplasia [41]. Our results show that patients who had met the diagnostic criteria for metabolic syndrome were not only more likely to have more acute prostatic hyperplasia, but also to have more troublesome Lower Urinary Tract Symptoms (LUTS) associated with prostate enlargement.

Lifestyle and diet are important factors in the etiology of both metabolic syndrome and benign prostatic hyperplasia. An excessive abdominal circumference in relation to height, the result of excessive body weight, is the primary diagnostic criterion that must be considered in the diagnosis of MetS. A relationship between visceral obesity and the risk of BPH are widely described in literature [31,42,43,44,45,46]. However, there are also a few reports refuting the existence of this relationship [47,48,49].

In our study body weight and abdominal circumference did not differ significantly between BPH patients and controls. Similarly, Meigs *et al.* [47] found that obesity did not affect the symptoms associated with BPH. In a study by Burke *et al.* [49], 40–71 year old men with prostatic hyperplasia were subject to anthropometric measurements of their body and abdominal size did not affect the volume of the prostate measured by ultrasound, and did not affect the pressure in urine flow through the urethra. In addition, there are reports of a negative correlation between the plasma concentration of the Prostate Specific Antigen (PSA) and the volume of the prostate, and obesity [50,51].

However, a larger group of researchers confirm the existence of a positive correlation between the volume of the prostate and factors associated with body weight. Based on the Baltimore Longitudinal Study of Aging (BLSA) large cohort study, Parsons [31] developed a model in which the volume of the prostate is greater by 0.41 ml with a BMI increase of 1 kg/m^2^. The author also noted that patients with a BMI qualified by WHO standards [52] as “second degree obesity” (BMI ≥ 35 kg/m^2^) were at a 3.5-fold higher risk of BPH compared to patients with normal body weight (BMI < 25 kg/m^2^) [31]. Another group of researchers [43] conducted a meta-analysis involving 5403 patients with prostatic hyperplasia coming from different countries, divided into those with and without metabolic syndrome. Men with MetS had a much larger prostate volume compared to patients without MetS.

Among the diagnostic parameters which were analyzed in our paper in the context of MetS, only abdominal circumference and HDL were significant for the risk of BPH.

Men from Asia have a much lower incidence of benign prostatic hyperplasia than their Western European peers. Denis *et al.* [44] show that a vegetarian diet and the regular consumption of low-fat and high-fiber foods promotes slower prostate enlargement. Those authors point to the advantage of a diet rich in soy, flaxseed, whole grains, and fruit and vegetables as a factor preventing prostate hyperplasia. In addition to its estrogen-like action, components of plant food may interfere with the metabolism of steroids and inhibit the activity of tyrosine kinase and topoisomerase, necessary for the proliferation of prostate cells.

Analysis of carbohydrate metabolism in the studied groups of men showed that patients with prostatic hyperplasia eligible for surgical treatment were more likely to have type 2 diabetes. One of the first studies on the impact of metabolic parameters on prostatic hyperplasia was conducted by Hammarsten *et al.* [53], who observed a correlation between hyperinsulinemia and increased prostate volume. They put forward a hypothesis that the activity of the sympathetic nervous system is increased in patients with prostatic hyperplasia. Stimulation of the sympathetic nervous system results in the stimulation of α-adrenergic receptors in the smooth muscles of the bladder and prostate, which leads to their contraction. These changes aggravate the symptoms of BPH [54].

The levels of insulin, insulin-like growth factor, obesity [55] and diabetes [56] are considered to be independent risk factors for the development of BPH. Similar to our results, in the study by Nandeesha *et al.* [55] on 88 men, HOMA was significantly higher in men with BPH, and similar to fasting insulin levels (average: 237.4 pmol/L *vs.* control group 134.7 pmol/L, *p* < 0.001); the researchers indicated that insulin resistance may be an independent risk factor for the development of BPH. However, our research showed no significant differences in HOMA between patients with and without BPH.

A hypothesis about the association between insulin resistance and benign prostatic hyperplasia is based on the mechanism where the increased levels of insulin result in insulin resistance and thus increased levels of insulin-like growth factors (IGF 1). This is followed by the inhibition of hepatic secretion of insulin-like growth factor binding protein (IGFBP-1), an IGF 1 antagonist [57]. The reduced IGF 1 level within the prostate promotes prostate cell proliferation [58]. This hypothesis has been confirmed by Kleinberg *et al.* [59] where infusions of IGFBP 1 in mice with BPH resulted in a significant reduction in prostate weight.

The relationship between disorders of lipid metabolism and the pathogenesis of benign prostatic hyperplasia has been documented in several studies [60,61,62]. According to the European Society of Cardiology [63], the correct concentration of total cholesterol (ChT) in men is <190 mg/dL, low-density lipoprotein (LDL) cholesterol <115 mg/dL, high density lipoprotein (HDL) >40 mg/dL, and triglycerides (TAG) <150 mg/dL. There is no conclusive data on which lipid fractions are associated with the pathogenesis of prostatic hyperplasia. In our study patients with BPH had significantly lower levels of ChT, LDL and HDL, while TAG levels did not differ significantly between those with and without BPH. The physiological mechanism of these associations is not completely understood. Lipids are molecules, whose main role, aside from the one associated with building biological membranes in the cell, is sending signals in the cytoplasm. The direct relationship between the levels of lipids in plasma and BPH is difficult to determine. Authors emphasize the role of lipids in the regulation of cellular transcription [64], and their part in the development of insulin resistance [65]. Although researchers tend to focus on the effects of a high fat diet on the pathological prostate hyperplasia, shown in studies on animals [60,61,66].

In an experiment conducted by Ploumidou *et al.* [60], male Wistar rats fed a diet enriched with cholesterol (4%) and cholic acid (1%) demonstrated not only altered blood lipid levels, but were also characterized by a hyperplastic prostate. Pathological changes in the prostate’s structure could have been due to parallel changes in sex hormone concentrations. In a similar experiment, Rahman *et al.* [61] demonstrated that Sprague-Dawley rats that had received a high fat diet had a larger prostate gland volume and also experienced erectile dysfunction and urinary bladder hyperactivity. Escobar *et al.* [66] conducted an experiment consisting of assessment of the effect on the prostate of a diet rich in different types of fat. The first group of Wistar rats received large amounts of lard in their diet and the second group received linseed oil. The group fed with lard demonstrated a significant increase in prostate weight, whereas in the group fed with linseed oil it was reduced. Western blot analysis showed that the group fed lard had an increased expression of androgen receptors (ARs) and peroxisome proliferator activated receptors (PPAR-γ). The diet rich in unsaturated fats promoted a decrease in the expression of those receptors [61]. PPAR-γ receptors are important in the etiology of BPH due to their effect on the differentiation and maturation of adipocytes, and participation in the pathogenesis of insulin resistance in tissues [67]. When analyzing pathogenic factors contributing to the development of prostatic hyperplasia and escalation of its symptoms, one should not forget about hypogonadism, as a factor related to inflammation of the prostate gland. A study conducted on the animal model by Vignozzi *et al.* [35] demonstrates that animals fed a high-fat diet develop hypogonadism and MetS-related features. Such a diet has also effects on the occurrence of pathological changes in the prostate, such as inflammatory states, fibrosis and hypoxia of this gland. Studies confirm that a similar relationship can be observed in men with LUTS and BPH [68]. It is also worth mentioning that a factor which plays an immunological role in the regulation of the inflammatory state of the prostatic stromal cells is dihydrotestosteron (DHT).

Ozden *et al.* [69] investigated lipid parameters in patients with diagnosed BPH. The analysis shows a relationship between MetS in patients with BPH and lower-than-standard HDL level and a higher TAG. Their experiment suggests that atherogenic dyslipidemia, which is one of the diagnostic indicators of MetS, has an impact on the growth of the prostate. Zhang *et al.* [70] examined Chinese men, and similar to our study they found that metabolic syndrome and low HDL levels may be considered risk factors for BPH. Similar conclusions were reached by Nandeesha *et al.* [55] who found that patients with prostatic hyperplasia had a much higher concentration of ChT and LDL and lower levels of HDL in plasma, compared to patients without prostatic hyperplasia. In that study the concentration of insulin in patients with diagnosed BPH significantly correlated with ChT and TAG levels.

Due to the aging of the population, both BPH and hypertension are found in a growing number of patients. It is estimated that these disorders occur simultaneously in about 30% of men [71]. BPH and hypertension are different diseases, although they share an etiology associated with the sympathetic nervous system. Our study showed no relationship between hypertension and prostatic hyperplasia. This is contrary to the results of Michel *et al.* [72] who studied men with clinical BPH symptoms. Hypertension in that study was defined as a diastolic blood pressure over 90 mm·Hg, diagnosed hypertension or administration of pressure-lowering drugs. The study showed a statistically significant relationship between the occurrence of hypertension and prostatic hyperplasia.

Experts in the field of hypertensiology and urology have created a system of synergistic treatment of both diseases with the use of α_1_-blockers [73]. For many years these blockers were considered drugs of choice for use in hypertension with concomitant BPH. This situation changed in 2000 following publication of the results of the Antihypertensive and Lipid Lowering Treatment to Prevent Heart Attack Trial (ALLHAT) [74], showing that the use of those drugs could cause heart failure and other cardiovascular diseases.

## 6. Conclusions

Finding the relationship between metabolic disorders and benign prostatic hyperplasia has important therapeutic implications in the treatment of diseases of aging in men. The conducted studies confirm the importance of carbohydrate and lipid disorders in the etiology of BPH. In the study presented in this article, statistically significant relationships between BPH and the diagnostic parameters of the metabolic syndrome were demonstrated. These results indicate to the necessity of the modification of the lifestyle, taking preventive measures in diabetes, and evaluation of lipid metabolism disorders. It is recommended to assess symptoms that may suggest BPH (as a manifestation of LUTS) in men over 50 years of age with diagnoses of metabolic disorders (including MetS), and provide them with specialist urological care in order to prevent surgical treatment of the prostate. The results of our study indicate the need for further research and analysis of the impact of metabolic disorders in the etiology and course of benign prostatic hyperplasia.
